# Extended producer responsibility’s effect on producers’ electronic waste management practices in Japan and Canada: drivers, barriers, and potential of the urban mine

**DOI:** 10.1007/s43621-023-00124-y

**Published:** 2023-02-13

**Authors:** Mika Kaibara Portugaise, Lára Jóhannsdóttir, Shinsuke Murakami

**Affiliations:** 1 University of Iceland, Reykjavik, Iceland; 2 The University of Tokyo, Tokyo, Japan

**Keywords:** Electronic waste, E-waste, Extended Producer Responsibility, Urban mine, Drivers, Barriers

## Abstract

Electronic waste is the fastest-growing domestic waste stream globally, continuously outstripping projections. With increasing ubiquity of complex computing, many non-renewables are contained in end-of-life electronics, creating a vast urban mine, potentially hazardous, depending on treatment. The aim of this study is to compare how Extended Producer Responsibility (EPR) policy is applied in two case countries, Japan and Canada, the practical implications of EPR policy design on producer operations, and how EPR affects electronic waste management improvements in each case. These cases share international obligations for electronic waste management but employ contrasting EPR policies. These policies are widespread in both cases, yet are not presided over by larger, regional obligations. Therefore, country-level interviews with electronic waste management stakeholders focusing on how EPR regulation affects producer practice were conducted. The physical application of EPR, as seen in Japan, drives design changes by producers intending to simplify downstream treatment, while financial responsibility in Canada, creates greater concern with cost-savings for producers, complicating end-of-life processing. EPR implementation, along with specific geographical factors, also create contrasting resource recovery results between countries. Regulation primarily drives EPR implementation in both countries, which is consistent with the literature. This study presents new drivers and barriers, namely pre-emptive legislation, and no incentive to improve, classifying the Japanese and Canadian systems as suffering from externalities on an insular system, and lack of harmonization, respectively. This research addresses a gap in comparative studies across regions of physical and financial EPR effects on producer practice.

## Introduction

Waste electronic and electrical equipment (WEEE or e-waste) is experiencing the most rapid growth of the domestic waste streams, but it also contains incredible potential for emissions mitigation, economic growth through resource recovery, and movement towards closed-loop product-to-waste-to-product systems [1]. As a waste stream, WEEE presents a unique case for its dichotomous hazard to human and environmental health and its high potential for greenhouse gas (GHG) mitigation through resource recovery [2, 3]. Although 78% of the global population is beholden under legislative mandate on the collection and recycling of e-waste, less than 18% of total e-waste was reported to have been properly documented, collected, and treated in 2019, a disparity projected to cost the global economy more than $57 billion USD in unrecovered raw materials [1]. There is also an associated social cost, both in human and environmental harm to those who mine or scavenge e-waste outside the formal, legal frameworks in place [1]. Consequently, current regulation at both international and national levels employs Extended Producer Responsibility (EPR) as a tool to task electronic and electrical equipment (EEE) producers with the management of WEEE. EPR is defined by the Organization for Economic Co-operation and Development (OECD) as a policymaking method which assigns responsibility to producers to treat, manage, or dispose of products in the post-consumer stage of life [4].

This study aims to compare how Extended Producer Responsibility policy is applied in two case countries and the practical implications of EPR policy design on producer operations, as well as what motivates or impedes WEEE management improvement in those countries. By focusing on two types of EPR: physical responsibility and financial responsibility; and highlighting examples where each is implemented as a system, we aimed to compare and examine the differences that arise from applying theoretically different models of EPR to e-waste management practice, from the producer's standpoint. We chose Canada and Japan as case studies, because they share the same OECD position as Category I countries and thus the same international legal obligations for WEEE management, yet they have taken distinct approaches to the administration of EPR, with Japan utilizing Physical Producer Responsibility and Canada Financial Producer Responsibility as the compliance mechanism of choice. Both also generate a very similar per capita rate of e-waste—in 2019 this was 20.4 kg per capita in Japan, and 20.2 kg per capita in Canada [1].

Physical Producer Responsibility refers to producers overseeing and implementing management practices for their e-waste, while financial Producer Responsibility is when producers do not manage operational aspects of EPR requirements but cover the costs to do so [5]. To achieve the primary objective, it addresses the application of physical and financial EPR regulations through their impacts on producers to determine both internal and external drivers and barriers for EPR.

The research as defined by the objectives has three important categories: (1) the state of country-level e-waste management through application of EPR (2) the potential of the urban mine, and (3) the drivers and barriers faced by producers. Therefore, the research questions are presented:

1. How does physical Extended Producer Responsibility influence producer behavior when compared with financial Extended Producer Responsibility in Japan and Canada’s WEEE recycling management schemes?
2. How does the difference between Japanese and Canadian systems affect the post-collection cycle of resource recovery and the viability of urban mining from e-waste?
3. What are the drivers and barriers to producers implementing Extended Producer Responsibility in the cases of Japan and Canada?

To address these questions, the two case countries, Japan and Canada, are explored and compared. Section 2 presents a review of current and relevant literature while Sect. 3 addresses the research methods applied. Section 4 presents results for each case as well as a comparison between them, and Sect. 5 synthesizes these results into a discussion answering the research questions. Finally, Sect. 6 will present final conclusions and suggestions for future study.

## Literature review

This section provides a summary of related topics to the proposed area of study based on recent literature in order to contextualize the relevance of this research. This includes the Global State of WEEE Management, including a brief history of EPR and informal recycling operations, as well as the urban mine and upstream vs. downstream mitigation.

## Extended producer responsibility

Extended Producer Responsibility (EPR) was first discussed in the 1990s by Lindqvist and Lidgren at the Swedish Ministry of Environment [6], and subsequently popularized in the widely referenced EPR Guidance Manual for Governments published by the Organization for Economic Co-operation and Development (OECD). In theory, EPR shifts the management burden of waste products away from municipalities to return products to producers at End-of-Life (EoL), intending to lower the public sector’s financial burden while pressuring producers to create easier-to-recycle products [7]. There are very few studies which compare the effects of physical and financial responsibility on WEEE management. One recent study done by Rau et al. compared physical and financial EPR in Taiwan and South Korea but focused on a single product and utilized a social welfare value model to assess effectiveness. Additionally, few direct cross-regional studies exist—instead, studies more commonly analyze global or regional EPR policies at a summative level, without directly comparing how EPR is implemented in different countries [8–11]. Furthermore, no current studies have explored physical and financial EPR in e-waste using primary data from the producer perspective. This research therefore seeks to address the literature gap in how producers in two regions are practically affected by the application of physical and financial EPR regulations. The drivers for EPR have been studied previously, both through review of available literature [12, 13], and through primary data at a national level [14, 15]. Barriers to implementation of EPR have also been discussed in the literature [4, 16]; however, both drivers and barriers have not yet been presented in the same format, both from primary data and compared against the relevant literature, one outcome of this study.

## Current state of waste electrical and electronic equipment management

There is no global definition of e-waste/WEEE, varying between countries and regions, and trends toward ubiquitous computing are constantly expanding the pool of post-consumer items that should be classified as WEEE (Khan 2016). For example, laptops, home appliances such as refrigerators or air conditioners, cell phones, medical equipment, and printers, are a small cross-section of the wide variety of items that can be classified as EEE during their usable life, and WEEE when disposed of. Another central acknowledgement is that the regulating body for e-waste is the Basel Convention, which broadly covers the Control of Transboundary Movement of Hazardous Waste and their Disposal. The Convention presides only over the transboundary movement of WEEE due to hazardous constituent parts, while methods of recycling, WEEE management, and EEE design and composition are not internationally governed [11]. Finally, the accelerating rate of generation of WEEE globally, especially as developing countries begin to generate larger amounts of domestic e-waste, and the expectation that globally, electronic waste will become more strictly regulated, indicate that understanding how EPR implementation can be practically improved is apropos to the current state of global WEEE management study [11].

The 85% of global e-waste which is reportedly mismanaged can disperse into three disparate unclassified WEEE streams: (1) disposal with regular municipal solid waste ending up in landfill (2) export as used electronic and electrical equipment (UEEE) and (3) illegal trade to informal recycling operations [17]. Streams 2 and 3 are distinctly linked, as lack of clear governance structure results in large concentrations of e-waste handled through black market trade to informal recycling streams. These streams are characterized by recycling practices not sanctioned or regulated by any legislating body and are largely concentrated in countries with less stringent environmental protection policies; however, the origin of these flows is widely acknowledged to comprise developed countries, therefore the effective application of EPR in developed countries -such as the cases of Japan [18] and Canada [8]—has potential for remediation of illegal flows [19–21].

The dichotomous nature of WEEE is highlighted in its potential value as a secondary stream for natural resources of dwindling primary supply. It is also, in some cases, more energy- and cost-efficient to extract precious metals from WEEE than from virgin mining processes [22]. This interplay between decreasing availability of primary stream- and increasing secondary stream-resources, particularly rare earth metals, was first discussed in the literature in the 1980s [23]. The term ‘urban mine’ was coined to describe areas of industrial products concentrated on the earth’s surface. The definition of the urban mine has since expanded to more broadly describe the global potential of resources which have already been mined through primary streams and are now contained within used products and waste [24]. The concept of the urban mine fits into a larger discourse about sustainable development and creating circularity between EEE and WEEE by moving towards Circular Economy [8]. Particularly, current systems are said to underperform in resource recovery potential due to limitations of recycling technologies [25, 26], yet the design focus of EPR policies may contribute to whether innovation is encouraged in the recycling field [26]. Therefore, improvement of resource recoverability through both limitation of exported EoL WEEE and improvement of domestic recycling capabilities is critical—in particular, a 2016 study showed that only PHS (Personal Handy-Phone System) terminals and cellular phones could be economically recycled in Japan under current conditions [18].

The intended mitigation of e-waste’s harmful lifecycle through regulation can be understood in two dimensions: (1) Upstream; The design and production phases, by reducing hazards and applying innovative measures which either extend the product’s life, or de-toxify the inevitable waste resulting; (2) Downstream, or post-discard, managing environmental impacts of final disposition. EPR is seen as having the potential to both drive greener design and manage the entire lifecycle of a product, therefore driving the electronics industry toward Circular Economy goals [25]. The literature, however, suggests that EPR may be better suited to improving downstream systems, such as collection into effectively managed recycling schemes, despite the arguably greater impacts of upstream mitigation [8, 27]. For instance, certain design-stage decisions, like light-weighting, may have upstream benefits such as lower material inputs and therefore potentially lowered transport emissions, however, these changes may negatively affect ability to recycle at End-of-Life (EoL) [28]. As a result, doubts have been raised about the efficacy of EPR in improving WEEE management, especially in the upstream—multiple studies have indicated that producers see EPR as an obligation to meet, rather than an opportunity to improve management practices or product design [25, 29]. Thus, how to integrate EPR policy with producer practice more holistically to address environmental outcome deficiencies and the perceived upstream–downstream dichotomy is a prescient topic [25, 26].

## Methods

This case study is based on the Grounded Theory Method [], with data collected through the use of explorative expert interviews [30]. The intent is to establish a basis for future research through comparative analysis of data gathered systematically, but inductively—by which theory is established through interpretation of data as a paradigm to understand certain phenomena [31]. This study aligns with the constructivist approach of aiming to understand participants worldview through qualitative data analysis per Gubrium et al. [32].

### Case selection

The case countries were selected for the following reasons:

1. Japan and Canada are both OECD members and signatories to the Basel Convention but have not ratified the Basel Ban Amendment. In short, they carry the same obligation for management and compliance at an international level.
2. Japan’s system is one of the clearest and oldest examples of physical producer responsibility in practice, while Canadian policies are largely implemented through financial producer responsibility.
3. Both countries showed early interest in adopting EPR legislation to govern WEEE management.
4. Both countries have strong industry groups who have played a prominent role in regulation design and implementation.

Japan is a geographically small, population-dense country with the world’s third largest economy by GDP (World Bank 2022). In contrast, Canada is the second largest country by landmass, but the population density is extremely low in most regions, with approximately 66% of the population living within 100 km of the U.S.-Canadian land border (Statistics Canada 2017). While the Japanese electronics production market share has fallen significantly from a 21% share of the global market at its peak, it is still estimated to contribute significantly to electronics production at 11% of the global market (JEITA 2020). There is no publicly available data about the size of Canada’s global electronics production market share, but based on a 2020 study, Canada was responsible for as little as 0.02% of globally generated e-waste [33]. These two cases were selected due to their differences in application-type of EPR, but also because they are similar in that they are both considered developed OECD countries, both have widespread e-waste management policies in place (unlike the United States of America) but are not part of a larger regional framework (like EU countries), and generate a very similar amount of per capita e-waste [1]. There is also a difference in central and local governmental relations for policy management—Japan’s system is highly centralized in terms of policy implementation, while Canada has distinct policy for each province, although these can be practically categorized together, with the differences being more implementation- rather than practice-related.

A case study approach [34] with a comparative focus between two cases was selected due to the relatively low number of available studies, the lack of comparison across regions despite ubiquity of the issue, and the discrepancy between legislative targets and actual reported data [8, 35]. Furthermore, negative by-products and side effects of e-waste management in its current form and the as-yet untapped potential of the urban mine lend relevance to this field of study across multiple disciplines and approaches [1, 10].

### Data collection

Data collection for this research comprised interviews, with interviewee selection and the framework developed based on the explorative expert interview, according to Flick [30]. The aim of utilizing explorative expert interviews, specifically, is exploring a new area of study to arrive at a hypothesis or a thematic structure [30]. In this case, to better understand how differing EPR regulations across regions affect resident producers, especially the same producers acting in different regulatory markets, and stakeholder dynamics and dependencies that result in EPR systems. The research questions focus on producer organizations; however, it has previously been noted that the public policy process remains tied to the amelioration of societal issues, and this is not naturally provided in the marketplace [36]. This supports the findings of the literature, in which external drivers were shown to be more influential on companies ‘ implementation of EPR policies, with regulatory or legal mandate being the top driver in both studies [12, 15].

Tables 1 and 2 identify the interviewees by a randomly assigned number or a letter in order to protect the privacy of participants, identifying them only by stakeholder group. Japanese interviewees are identified by a J-1, J-2, J-3, etc., and Canadian interviewees by C-A, C-B, C-C, etc. A total of 19 interviewees participated over the course of 15 interviews, including 8 in Japan and 9 in Canada, representing both nationwide interests and specific interests of two regulated provinces. It was noted that local environmental Non-Governmental Organizations (NGO) are not active on a wide scale in this field in either Japan or Canada at this time (Interviewee C-G and Interviewee J-8). Table 3 provides a comparison between representation of stakeholder groups in interviews.

**Table 1:** Japanese interviewees

Interviewee	Stakeholder group
J-1	Producer
J-2	Collector
J-3	Producer
J-4	Industry group
J-5	Government
J-6	Producer
J-7	Recycler
J-8	Academia/policy

**Table 2:** Canadian interviewees

Interviewee	Stakeholder group
C-A	Industry group
C-B	Academia
C-C	Government
C-D	Certification body
C-E	PRO/recycler
C-F	Consultant
C-G	Regulator
C-H	Regulator
C-I	Producer

**Table 3:** Comparison of stakeholder representation between interviewees in Japan and Canada

Stakeholder group	Japan	Canada
Industry group	1	1
Producer	3	1
Government	1	1
Regulator	0	2
Academic	1	1
Collector/recycler	2	1
Consultant	0	1
Certification body	0	1

Interviews averaged one hour but ranged from 30 to 90 min. Interviewees were provided an interview framework (see Appendix for standard interview frameworks—then tailored to each interviewee) in advance, but these were typically used as guides during the actual interview, conducted in a semi-structured manner, noted to be a relevant method for understanding expert viewpoints [30]. The interview framework was tailored slightly to each interviewees’ expertise and stakeholder group but was designed to provide comparability across interview responses. The interviewer used follow-up and probing questions to address topics identified in the interview framework and clarify points brought up during discussion.

Interviews for the Japanese case were carried out between July and August of 2022, and while the Canadian case interviews were mostly completed between March and June 2022, a few were completed concurrently with Japanese case interviews. Interviews were conducted in a combination of online and in-person interviews, in response to the ongoing COVID-19 pandemic. All online interviews were done in Microsoft Teams, recorded, and transcribed natively. In-person interviews were recorded on a handheld recording device and then transcribed manually. Interviews were conducted in English, where possible, to create consistency; however, this was not possible for four of the Japanese interviews. Three of these were conducted with the assistance of a second-year Master’s student at The University of Tokyo, under the supervision of Professor Murakami, who provided real-time translation as well as translated transcripts and question frameworks. The final Japanese interview was completed with the assistance of a professional translator, translating verbatim between Japanese and English (and vice versa) in real-time, therefore, the transcript was created by the researcher in English.

### Data analysis and processing

To establish credibility for the researcher’s analysis and interpretations, two iterations of member checks were conducted, in which interviewees are provided with transcripts of interviews to assess the accuracy of transcription [37]. The first was for the interview transcripts, before analysis, and the second was for the final draft of this study, prior to submission. After conducting the first round of member checks, transcripts were transferred to MAXQDA2020 for data analysis. The software was used to group transcripts according to case country and for the coding process. This research followed the methodology of Grounded Theory Coding, and follows the steps defined therein according to Corbin and Strauss [38]: open coding; axial coding; and selective coding.

A combination of in vivo and constructed codes were developed based on themes arising from the source material (interviews) and the review of literature [30] through a combination of inductive and deductive approaches. Main themes relevant to the research topics were applied as constructed codes, including but not limited to: *physical responsibility; financial responsibility;* and *resource recovery*. Drivers and barriers emerged through open coding. Concurrently, the use of in vivo coding uncovered unexpected themes to show how policy/academic literature and business practices diverge in priorities and language. A sample of these are *light-weighting; market size;* and *design for recyclability.* In this study, the initial open coding round produced codes categorized as drivers and barriers, which were then compared and classified further as external or internal. Based on these classifications as external or internal drivers or barriers, further open codes identified specific recurring instances across the case interviews. This new collection of codes was re-organized along the axial coding paradigm model [38] to define emerging phenomena.

Throughout the coding steps, and elucidated during the axial coding phase, one theme emerged repeatedly in analysis of interviews for the Japanese case, whose story can broadly be contextualized as *effects of externalities on an insular system*. The externalities here referred to can also be called external factors, which affect how Japanese producers are able to implement EPR policy but which they are unable to directly change. In contrast, the central theme which emerged in the Canadian case was distinctly a *lack of harmonization*.

## Results

This section addresses the findings for each case separately, followed by a section on the comparison case, before presenting the top drivers and barriers arising from the study.

### Japan

Japan’s current E-waste policy comprises three acts. The first is the Act on Recycling of Specified Kinds of Home Appliances (Act No. 97 of 1998), also referred to as the Home Appliance Recycling Act, promulgated in 1998 and enforced from 2001. Second, the more recent Small Home Appliance Recycling Act (Act No. 57 of August 10, 2012) covering small WEEE. Finally, the Act on the Promotion of Effective Utilization of Resources (Act No. 48 of April 26, 1991) regulates personal computers as part of a voluntary producer take-back and recycling scheme [39]. This study focuses on the Home Appliance Recycling Act, as the other two Acts are not fully compatible with the research objectives for the following reasons: The Small Home Appliance Recycling Act is a voluntary and non-EPR system, while the Act on the Promotion of Effective Utilization of Resources is a semi-voluntary EPR scheme.

**Fig. 1:**
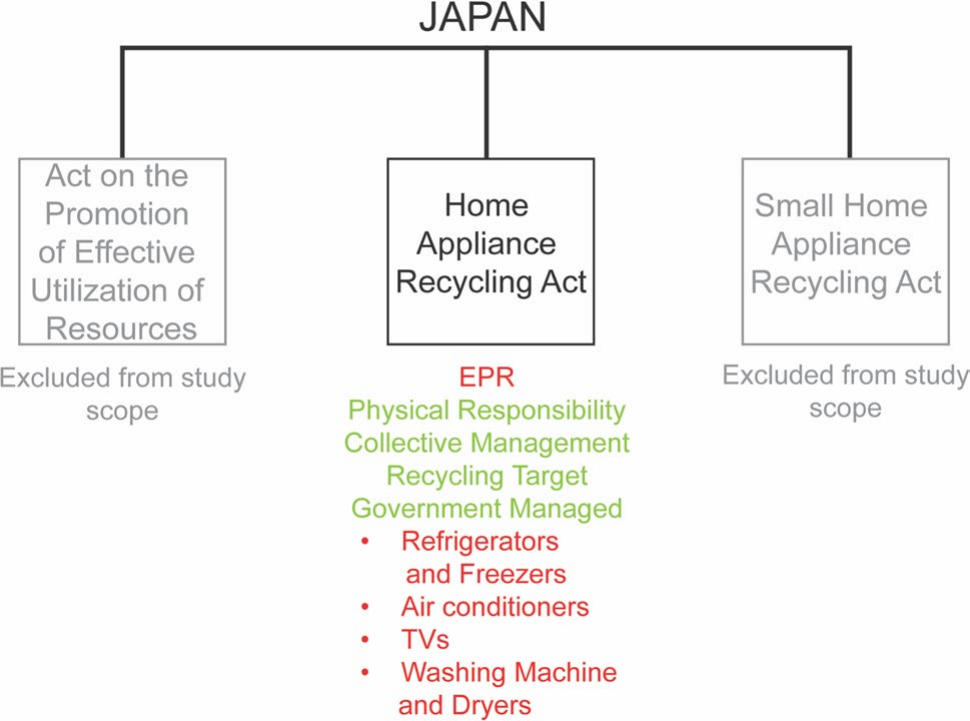
Relevant WEEE regulation systems: Japan—synthesized by authors

In Japan, the Home Appliance Recycling Act operates under a physical responsibility requirement [40] as seen in Fig. 1, which also shows obligated categories in red and regulation characteristics in green.

#### Home Appliance Recycling Act (Act No. 97 of 1998)

The Home Appliance Recycling Act was necessitated by the difficulty in managing disposal of home appliances as they became common household items—particularly due to the presence of re-usable resources [41], as well as consumerism driven by the economic bubble of the 1980s [42]. There was also an increase in public awareness and therefore concern about the depletion of the ozone layer due to chlorofluorocarbons (CFCs), and the hazards of other materials present in appliances [41].

Four appliance categories are obligated under this regulation: air conditioners; refrigerators; televisions; and washing machines. The law is one of the earliest examples of an EPR approach to e-waste management, in which manufacturers/producers are responsible for recycling their products at EoL. Japanese laws apply responsibility for waste management processes by clearly defined stakeholder roles. In this case, manufacturers bear physical responsibility for recycling products effectively; retailers bear responsibility for collection and transfer to recyclers; and consumers bear the cost of disposal in the form of a collection and recycling fee [42]. Japanese regulation does not dictate an annual collection target which producers need to meet; however, they must report their collected tonnage, of which the legally obligated recycling target percentage must be met in the form of re-manufactured or resold resources [42]. There is also an aspirational collection target set by the government, but no penalty is set for stakeholders who fail to meet it [43]. So, while the amount of collected WEEE may vary year-to-year, the recycling target must be met every year, according to the actual weight of e-waste units entering the recycling stream. The fee system is how manufacturers fund their recycling operations, and the cost is determined by the type of appliance and the manufacturer [42].

The Home Appliance Recycling Law applies statutory recycling rates for its four obligated categories, see Table 4 [43]:

**Table 4:** Japanese Home Appliance Act recycling targets [43]

Appliance	Original recycling target (%)	Current recycling target (%)
Air conditioners	60	80
Televisions (CRT/Flatscreen)	55/50	55/74
Refrigerators/Freezers	50	70
Washing machines/Dryers	50	82

#### Policy implementation

Manufacturer recycling for the Home Appliance Recycling Act is implemented by two alliances of appliance producing companies named Group A and Group B. The groups were formed to provide competition in the appliance recycling industry, and as such, they produce approximately the same number of appliances by unit. Both groups have a dedicated recycling management company, which represents all allied companies, coordinates between key players, and is responsible for data reporting and analysis [42].

Across the spectrum of waste electronics regulated in Japan, illegal collection, dumping, and export are the most frequently reported contraventions of the system. These have been discussed extensively in the literature, along with potential methods for amelioration [18]. There are also studies on how resource recycling may contribute to achieving environmental outcomes [44], with one study showing that for three out of the four regulated categories, over 50% of GHG emissions could be mitigated in comparison to virgin production [2].

For the Japanese case, the main constructed codes applied for data analysis were *physical responsibility, regulation, resource recovery,* and various drivers/barriers that presented in the literature: *illegal collection/export/dumping, cost, increased competitive advantage,* etc. *Financial responsibility* did also arise in discussions comparing Japan’s various legislations, but only in one interview. *Illegal collection/export/dumping* was a constructed code, but was also discussed in every interview, showing its pertinence for this study. Some constructed codes which had less relevance to interview findings than expected were *collective system/free riders, environmental protection,* and *ineffective targets.* In the case of the last one, the target system was not much discussed—they have not changed much over the years and producers regularly meet their obligations. As for in vivo codes, *e-commerce regulation, design for recyclability,* and *commodity fluctuations* represented much of the interviewees’ discussion. These account for some of the most current pressures on producers and emphasize producers’ physical responsibility in the e-waste management scenario in Japan.

The axial coding phase classified phenomena and their causes and effects which presented across previous coding phases to create a comprehensive picture of the case, refined during selective coding. Thus, the central theme for the Japanese case, *externalities on an insular system* represents multiple dimensions, drawn from the axial coding phase, namely system externalities creating compliance challenges for physical producer responsibility, especially due to the stakeholder relationship system, externalities challenging resource recovery, and externalities creating both drivers and barriers for producers. Illegal collection, dumping, and export is the largest current and historical concern in the Japanese system (Interviewee J-1, J-5, J-8), and very much in line with the overall global concerns about transboundary movement of e-waste.

### Canada

Canada has no federal-level e-waste legislation beyond compliance with Basel-related obligations, therefore e-waste management is regulated at a provincial level, resulting in a lack of consistency across policies [45]. Currently, of the ten provinces, all have established WEEE management frameworks, and of these all base their systems on EPR, excepting Alberta, which has a government-run system with a government-associated, but independent, regulator. The cost of recycling is borne by consumers in the form of a point of purchase handling fee in all provinces, but the amount charged, and whether producers are individually or collectively responsible for WEEE take back and management is province dependent. Under this system, Canadian consumers bear most of the financial burden of responsible e-waste management. In addition, the landfilling of WEEE is banned in only three provinces; Prince Edward Island, Newfoundland & Labrador, and Nova Scotia [33].

Ontario passed new legislation in 2016, titled the Resource Recovery and Circular Economy Act, S.O. 2016, c.12, Sched. 1, under which the Electrical and Electronic Equipment section: O. Reg. 522/20 came into force on January 1st, 2021 [46]. Under this new regulation, Ontario is the first province or territory to implement an Individual Producer Responsibility (IPR) model, by which all WEEE management stakeholders in Ontario must be registered [46].

**Fig. 2:**
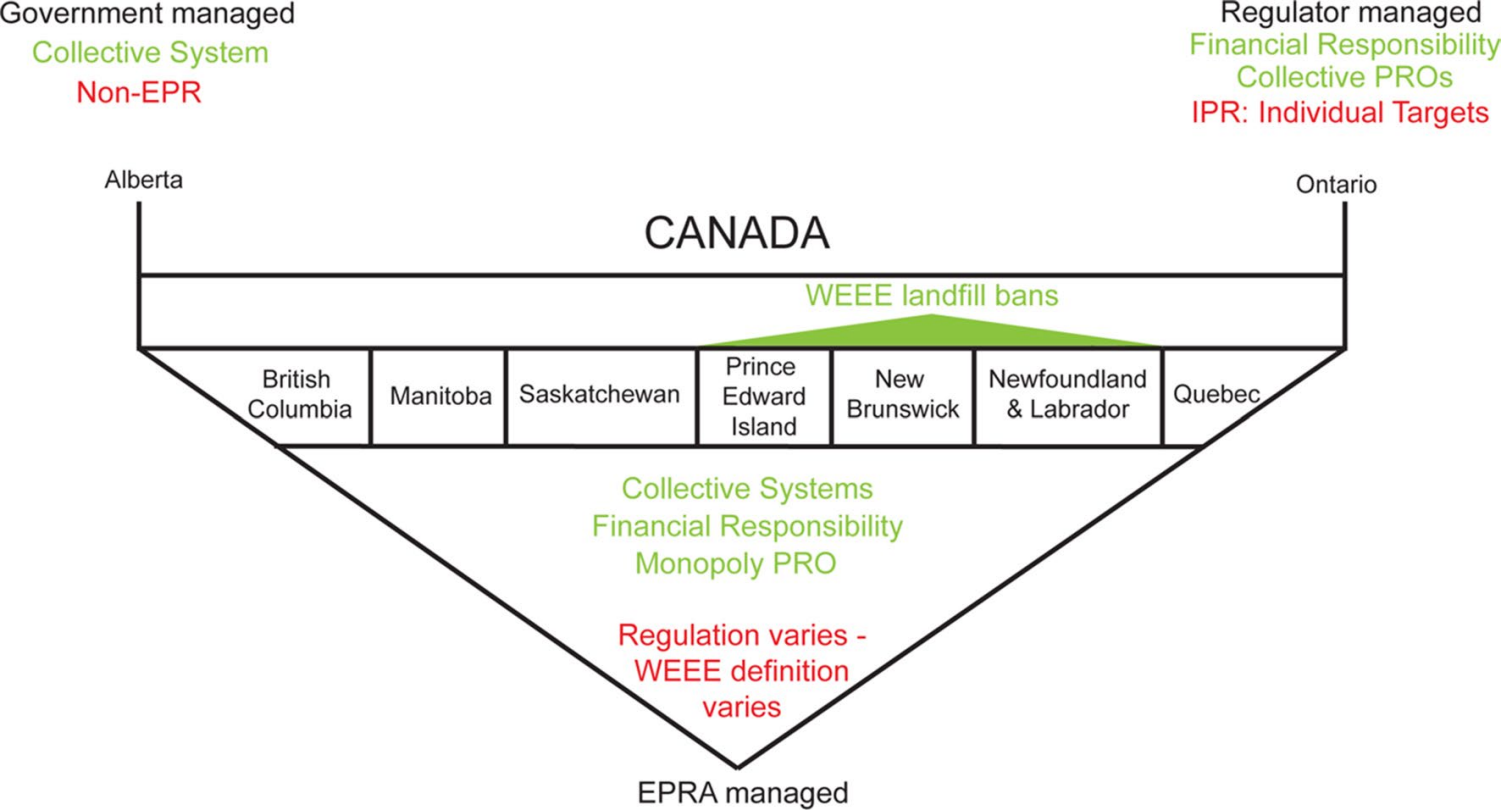
Relevant WEEE regulation systems: Canada—synthesized by authors

Eight of the ten provinces operate under financial responsibility EPR systems run by a primary PRO, Electronics Product Recycling Association (ERPA) [33], see Fig. 2. The red text defines obligated scope while green text covers the system characteristics. The remaining two provinces are Alberta—operating a government-run system, which in practice functions similarly to other provincial EPR programs—and Ontario—Canada’s first IPR system, managed by an independent regulator. Although the compliance and regulation mechanisms differ between provinces, Canada’s programs generally follow financial responsibility in practice, and their practical implementation is similar enough to be compared as one scheme and a counterpoint to the Japanese case [33].

Canadian regulation as well as the definition of what constitutes e-waste varies across provinces, creating a system which, while widespread, suffers from a lack of harmonization and data transparency [33]. Furthermore, the methods of calculating obligated collection rate can vary from province to province. Finally, the fact that there are now three distinct regulating bodies in Canada calls into question the possibility of harmonizing data and policy intent across the country in order to maximize environmental performance of the system [33].

In the Canadian case, constructed codes included *financial responsibility, regulation, ineffective targets, consumer awareness, education, corporate image.* In this case, consumer awareness and education were brought up only a few times, but were not key points of discussion, even though *corporate image* was the third most discussed driver. As for in vivo codes, despite the three systems in Canada, these proved to be universally important: *lack of harmonization, lack of data, market size,* as well as *light-weighting* and *recycling over reuse.* These in vivo codes, in combination with the constructed codes which were relevant express the Canadian system’s focus on EoL concerns and management of cost for non-resident producers.

Based on these findings, the axial coding phase and subsequent selective coding phase point to a lack of harmonization as the central theme of the Canadian case. This aligns with available literature and seems to follow global trends: specifically, the lack of a universal definition of e-waste. This has created difficulty in comparing performance across regions, and in fact, even across Canadian provinces. Every, interview done in Canada addressed two themes either directly or indirectly. The first was lack of harmonization, and the second was the size of the Canadian market. The Ontario program was specifically designed to provide producers with flexibility in how they meet their management obligations (Interviewee C–C). However, in practice, the Canadian system generally employs FPR, with a network of Producer Responsibility Organizations (PROs) operating the systems on behalf of producers. The lack of harmonization relates to the stated research question themes in the following ways: challenges created by the practiced responsibility system: FPR; how cost affects resource recovery/urban mining potential, and the drivers and barriers producers in Canada.

### Extended physical producer responsibility system performance

The Japanese system does not have an independent external regulator, as seen in the Canadian schemes, all three of which have an organization responsible for the setting of targets and monitoring of program performance which is not directly managed by the government. Conversely, Japanese governmental organizations Ministry of Environment and Ministry of Economy, Trade, and Industry set policy and recycling rate targets for producers and perform regulatory functions (Interview J-5). They also have a historically close relationship with domestic producers, reflected in the fact that policy changes are developed in collaboration with industry stakeholders in advance of implementation. The concept of responsibility role-sharing is central to the Home Appliance Recycling Law (Interviewee J-4). At the time of implementation of the law, domestic manufacturers produced almost all stock of the mandated four appliances (air-cons, fridges, TVs, washing machines), so physical producer responsibility was deemed an effective policy to tackle both upstream and downstream management. This was described by one interviewee as:

*The development of new recycling technologies and the review and efficiency of the recycling process will reduce the cost of recycling and allow recyclers [producers] to reap greater profits. By introducing such a mechanism, incentive to improve the current situation of recyclers continue to work, reduce the fee burden on consumers, and establish an economical system (J-5).*

Collaboration with industry is a long-standing tradition of the Japanese regulatory system, but also one that makes physical EPR particularly difficult to implement in light of challenges created by the externalities of globalization—such as decreasing domestic producer market share and illegal collection and export (Interviewee J-8). In short, these factors, external to the existing stakeholder system hamper producers’ ability to effectively manage WEEE generated in Japan.

### Financial producer responsibility system performance

In Canada a discrepancy that producers and recyclers deem harmful to overall performance of EPR e-waste management systems was that all ten Canadian provinces are covered by EPR (or similar) e-waste management legislations, but only three provinces have regulated landfill bans on electronic waste (Interviewee C-E, C-I). So, while legislation holds electronic waste processing done within the legal system to a certain standard, individual market players like second-hand resellers or charitable organizations who are not defined as obligated parties under the regulatory hierarchy can landfill e-waste and potentially cause significant environmental harm in doing so (Interviewee C-A, C-E). One producer stated:

*There are a number of provinces which refuse to ban EPR obligated products from their municipal landfill sites. Which is fundamentally improperly treated waste as it has zero waste recovery and allows the possibility of contamination of the environment (C-I).*

Another finding from the interview process in Canada was how interviewees perceived effectiveness of EPR in providing quantifiable outcomes—whether environmental, diversion-related, or design for environment (DfE) progress. Several interviewees suggested that EPR’s strength lies in creating take-back obligation and improving EoL diversion from landfill, but that DfE and other pre-EoL concerns may be better addressed in different legislation, for example the European Restriction of Hazardous Substances Directive (reducing hazardous materials—upstream) in contrast to the WEEE Directive (EoL collection and management—downstream) (Interviewee C-A).

### Resource recovery

In order to address the second research question’s study of resource recovery and urban mine potential, a combination of factors form a strong foundation for the ongoing practice of resource recovery in the Japanese system. First, the legal obligation of the recycling rate, ensuring that once EoL appliances are directed into the legal framework, they will be effectively managed to final disposition (Interviewee J-5); second, the strength of Japan’s material industry despite its lack of natural resource stocks (Interviewee J-8); and third, the economies of scale required for recyclers to economically manage e-waste create incentive to improve collection (Interviewee J-2, J-7). Changes to the Basel Convention and other regulation on transboundary movement also create uncertainty about the future of import and export of commodities (Interviewee J-2) and decreasing reliance on external sources is a significant driver for improvements in resource recovery from e-waste across all stakeholder groups (Interviewees J-1, J-3, J-5, J-6, J-7). Another motivating factor is the use of recycled materials and recovered resources in manufacturing to contribute to carbon reduction goals (Interviewee J-8).

In short, resource recovery seems to be a well-developed industry in Japan, with sufficient market pressures to drive increased capacity without any specific legislation to encourage it. Policy trends are, however, shifting towards the recognition of natural resources and the recycling industry as complementary parts of a self-sustaining system (Interviewee J-8). The resource recovery market is reliant on the fluctuation in market price of commodities, with one interviewee noting that:

*Manufacturers are making efforts to absorb cost increase factors such as rising logistics and labor costs through DfE [Design for Environment] and corporate efforts. Reducing recycling charges is our goal. However, recycling charges are closely related to resource prices. If resource prices drop very low, the value of recycled resources will also drop (J-4).*

While producers can profit from reselling resources, they highlighted the concern that certain commodities, especially plastic, are subject to price fluctuations which result in excess recycled stock with prohibitively low market value. Concern was expressed that certain plastics may even fall foul of increasing legislative requirements on specific chemical substances of materials and thus need to be incinerated (Interviewee J-4). Additionally, because recyclers can be expected to have a relatively steady supply of recovered resources, they are under pressure to provide adequate value and amount to producers (Interviewee J-1).

Comparatively, the Canadian system underperforms in resource recovery. Two producer cost-saving strategies were identified as impacting resource recovery ability: light-weighting and eco-fees. Light-weighting describes the aim to decrease the weight of electronics through material changes, increased efficiency, or reducing the overall size and was described by a producer as a method to save on cost as well as transportation emissions (Interviewee C-I). But conversely cited as an area of concern by multiple other stakeholders, including regulators and recyclers, as light-weighting typically means an increase in plastic, reducing the value of recoverable materials at EoL (Interviewee C-H). This is a distinct limitation that the Financial EPR model implemented in Canada creates.

Industry also expressed concerns that complying with a variety of complex legislations creates additional financial burden that producers need to bear to participate in the Canadian market. This is especially true given that producers in Canada largely pay for EoL management, but do not oversee it themselves, so lack the same ability to benefit from improved resource recovery performance, as seen in Japan.

Therefore, the implementation of financial EPR in Canada negatively impacts resource recovery practice—producers’ primary business is production, not EoL or product longevity, which influences upstream decisions. One interviewee described this issue as follows:

*I think there would be a stronger case for EPR affecting the upstream if it were genuine EPR, where companies were at risk of having their profit reduced by some increment, right?...It would be a stronger signal and the economic incentive to whatever, make your products last longer, easier to collect, you know, easier to dismantle, easier to repair, all that kind of stuff, at least potentially (C-B).*

In exploring the differences between the cases, urban mining and resource recovery—both potential and need for improvement—were seen as a very current issue in Japan.

*It’s not an issue for the Japanese case anyway, they have to do it, and they have a strong incentive to do it…in that sense, having a huge material producing sector is a Japanese uniqueness (J-8).*

Yet often expressed as a topic of interest, but future direction in practice in Canada, with one interviewee expressing the sentiment that technology is not yet sufficiently advanced to viably recover all resources (C-A). In fact, the term urban mine is more widely used and understood in Japan (especially as it was coined there) but needed clarification for some Canadian interviewees.

This is likely tied to the respective natural availability of resource deposits, given that Japan has limited natural resources, but a large material industry, therefore requiring external inputs to function (Interviewee J-8). Conversely, Canada’s abundance of natural resources and prominent mining industry has the potential either to drive improvements in recovery technology (Interviewee C-A) or hinder progress if recovered materials are seen as overly competitive to primary resource markets.

### Drivers and barriers

While many externalities to the Japanese system present themselves as barriers to effective EPR management of e-waste, there are also cases of externalities driving EPR improvements. The physical responsibility of the Home Appliance Recycling Act has driven improvements in product design for easier recycling, with one producer stating that:

*We apply the same concept to small appliances, and our basic concept is that easy to recycle items or products are easy to make as well (J-6).*

While producers assert that these benefits have spilled over into items not obligated under that legislation, for example through reduction of screws used to assemble products (Interviewee J-1, J-4, J-6), this point is contested by recyclers, who mentioned that “*the law doesn’t force action, but only promotes recycling”* (Interviewee J-2) and *“manufacturers should design products in a way in which the disassembly can be done easily”* (Interviewee J-7)*.*

As producers also tend to be recyclers in the case of the four obligated appliance categories, easier recycling and improved resource recovery rates should lead to greater profit. While the legislation prevents producers from profiting from recycling fees, it does allow them to profit from the resale of materials recovered during recycling (Interviewee J-5). Japan’s second-hand market for appliance-type items has typically been very small, while there is some growth in the PC and smartphone industry this market tends to exist outside of any producer involvement (Interviewee J-8). While the size of the reuse market can be estimated based on available metrics (Interviewee J-5), there is no comprehensive data, so determining the effect on producers’ business practices is difficult (Interviewee J-3).

The limit of obligated scope is also both a driver and a barrier—a driver in the sense that this narrow focus has allowed producers and recyclers to create a highly effective system, with high recycling rates and proven Design for Environment (DfE) improvements to their product line-up (Interviewee J-1, J-4, J-6); but also a barrier to expanding the system and adapting to the externalities of the growing e-commerce and import market (Interviewee J-8). Furthermore, consumer pay-on-collection fee structure has been noted to be problematic, as it may encourage illegal dumping or encourage consumers to utilize illegal collection systems to avoid paying (Interviewee J-4). Described by one interviewee:

*One of the biggest barriers would be that there are people who violate the law, and it is very important that the used products are properly treated according to the proper recycling route (J-1).*

While the timing at which consumers pay the recycling and collection fee—in this case, at EoL collection—was originally internal to the system, it has been raised as a concern at each subsequent re-assessment of the law with the intent to change it (Interviewee J-8), but all stakeholders cannot agree on a change. Changing the fee structure can thus now be considered an externality as producers cannot change the fee structure without the consent of all stakeholders, and therefore, it has become a factor of Japan’s EPR implementation which is beyond their control.

In Canada, the ten provincial legislations can be loosely classified under three systems: The Alberta Recycling Management Authority (ARMA) manages a government-adjacent program, Ontario’s Resource Productivity and Recovery Authority (RPRA) manages the new IPR program (still a new system and therefore without much available data on performance), and the remaining provinces operate under EPRA-managed EPR legislations (Interviewee C-E). However, as mentioned, all three systems can be grouped as financial EPR systems in practice. Interviewees addressed this topic differently. For instance, government and regulators noted difficulty in EPR system comparison. One recycler expressed a desire for a consistent and global definition of e-waste but was against federal oversight of the e-waste management industry in Canada; and producers/industry expressed difficulty in complying with so many programs, exacerbated by the small size of the Canadian market—1.2% of the Global Market (Interviewee C-I).

Despite the breadth of regulation in Canada, there is a widely acknowledged lack of data on system performance. This was attributed to: (1) lack of reporting on the reuse market (Interviewee C-I); (2) lack of import/export data on used electronics in Canada, leading to inaccuracies in quantifying flows which are used to determine producer obligations (Interviewee C-F); (3) Industry protectionism of identifying data where producers refuse to provide public data on market share, and regulators therefore refuse to provide specific data on obligated targets and whether or not these are met (Interviewee C-A, C-D, C-G, C-F). As mentioned by one interviewee:

*EPR does not measure the import and export of used electronics and electronic waste. There is a decent attempt to measure new electronics important by regulators such as RPRA, however, even that is limited to obligated electronics and not made publicly available (C-F).*

Tables 5 and 6 show the various internal and external drivers which arose and were refined during the coding process for each case, as well as their frequency. The top three drivers and barriers for each case with be listed in order below, as well as classified as external [E]or internal [I]; referring to the whether the drivers and barriers are outside of producer control or within their ability to affect. The top three drivers for Japan were: (1) Regulation/Take-back Obligation [E]; (2) Resource Recovery [E]; (3) Reduced Cost/Financial Flows [I], but the top three drivers for Canada were: (1) Regulation/Take-back Obligation [E]; (2) Reduced Cost/Financial Flows [I]; (3) Resource Recovery [E] & Corporate Image [E].

**Table 5:** External and internal drivers: incidence of codes compared between Japanese and Canadian cases

	Japan	Canada
External drivers		
Corporate image	10	8
Environmental protection	7	7
Increased competitive advantage	12	2
Pre-emptive legislation	11	7
Regulation/Take-back Obligation	23	24
Resource recovery	20	8
Internal drivers		
Certifications	6	5
Design for recyclability	7	1
Management awareness	5	5
Reduced cost/financial flows	16	15

**Table 6:** External and internal barriers: incidence of codes compared between Japanese and Canadian cases

	Japan	Canada
External barriers		
Collective system/free riders	2	4
Commodity market fluctuation	5	0
Complex legislation	11	17
Consumer awareness	8	3
Downstream management	10	5
E-commerce regulation	6	1
Education	2	1
Fee-timing	4	0
Illegal collection (+ Export/Dumping)	12	3
Ineffective targets	5	11
Lack of data	7	13
Lack of harmonization	5	16
Limit of obligated scope	7	11
Recycling over reuse	1	9
Transboundary regulation	8	7
Internal barriers		
Cost	17	14
Lack of management commitment	2	0
Light-weighting	1	11
Market size	0	7
No incentive to improve	12	8
Producer competition	0	7

The top three barriers for Japan were: (1) Cost [I]; (2) Illegal Collection/Export/Dumping [E] / No Incentive to Improve [I]; (3) Complex Legislation [E], but the top three barriers for Canada were: (1) Complex Legislation [E]; (2) Lack of Harmonization [E]; (3) Cost [I].

### Comparison case

Table 7 shows a more in-depth comparison between the two cases, including types of EPR applied as well as system characteristics and legal requirements. In short, this study classifies the Japanese case as representative of physical EPR, and the Canadian case as financial EPR. Based on Table 7, representing the synthesis of both this study’s research and the literature, the lack of consistency across regions and even within each case is clear, so it remains extremely difficult to determine which combination of factors is most effective.

**Table 7:** Comparison of WEEE Management Systems in Japan and Canada synthesized by authors

Country	Japan	Canada		
Legislation	Act on Recycling of Specified Kinds of Home Appliances	Ontario: Resource Recovery and Circular Economy Act	Alberta: electronics designation regulation	British Columbia, Manitoba, New Brunswick, Newfoundland & Labrador, Nova Scotia, Prince Edward Island, Quebec, Saskatchewan
Act #/effective date	Act No. 97 of 1998 (2001)	Act S.O. 2016, c.12 Sched 1. O. Reg 522/20 (2021)	Alberta Regulation 94/2004 (2004)	BC Reg. 162/2020 (2007), C.C.S.M. c. W40 (2010), Regulation 2015–247 (2017), Regulation 85/12 (2013), N.S. Reg. 26/2019 (2008), R.S.P.E.I. 1988, c. E-9 (2012), Q-2 r.40.1, Ch. E-10.22 Reg 6 (2007)
Obligated scope	4 types: Refrigerators, Air Conditioners, Washing Machines (& Dryers), TVs	ITT/AV consumer goods	Phase I electronics (+ pilot program with a wider obligated scope)	Scope varies by province, generally EPRA Phase II electronics covered, at a minimum
Collection responsibility	Retailers	Producer via PROs or collectors	Collectors/PROs	Producer via collectors/PROs
Fee structure	Collection & Recycling fee levied on consumer	Eco-fees (determined by producer), or cost-per-tonne (by PRO)	Eco-fees (determined by ARMA)	Eco-fees (determined by regulator)
Fee timing	At EoL disposal	Point-of-sale	Point-of-sale	Point-of-sale
Recycling responsibility	Producers	Recyclers/PROs (producer-contracted)	Recyclers (designated by ARMA)	Recyclers (designated by EPRA)
Program management	Government	RPRA (regulator, non-profit)	ARMA (government-adjacent regulator)	EPRA (primary PRO)
Physical or Financial EPR	Physical	Financial	Financial	Financial
Collective or Individual Responsibility	Collective	Individual	Collective	Collective
Target systems	Recycling Target (legally obligated)	Collection Rate (legally obligated)	100% Collection Goal	Collection Goal (excepting Quebec, which has enforceable targets)

The Japanese and Canadian case results share several key factors: firstly, both cases have complex legislation which suffers from a limited obligated scope. In the case of Japan, this can be attributed to the different applications of responsibility across the three Acts, and in Canada due to a lack of federal harmonization. EU Directive 2012/19/EC serves as a point of contrast to these systems—since 2018 it has adhered to an open scope policy, by which all EEE except that explicitly excluded falls under WEEE EPR management requirements [47]. Secondly, what constitutes e-waste is not clearly defined, as regulation obligates by specific product, rather than any defining characteristic. Much discussed in the Canadian case was the difficulty in dealing with light-weighted designs at EoL, often paired with acknowledgement of the small size of the Canadian market, while the challenges of regulating e-commerce were discussed in both Canada and Japan. While the different pressures produced by PPR and FPR were discussed in each case previously, the comparison highlights a lack of ability for regional EPR regulations to exert true DfE pressure on global, non-resident producers.

In both cases, interviewees also expressed concern about the increased stringency of transboundary legislation on movement of waste. This was emphasized as potentially limiting to the legitimate flow of post-processed, pre-manufactured raw materials recovered from e-waste for re-integration into production streams. In both Canada and Japan, stakeholders mentioned that classification of waste vs. resource is currently ill-addressed in regulation, ignoring global market and resource dynamics, which may hinder the ability to reuse recycled resources or create negative environmental outcomes (C-E, J-8). Specifically discussed in the Japanese case:

*Another reason we are importing a lot from example, Thailand or Vietnam is because Japanese manufacturers have factories there, so they are the processing scraps coming out of the factory, so we import some portion from those countries, and it’s quite safe because the exporters are Japanese companies. Otherwise, it’s quite difficult to import for us, but for example Japanese smelters and other material industries try to increase the imports, partially because they are trying to do it to decrease carbon emissions (J-8).*

Finally, the seminal issue of the lack of a standardized definition of e-waste was raised extensively, with an interviewee commenting that:

*For example, with the Basel Convention, even the most recent round of negotiations just earlier this year, you know, they once again have been unable to create a universally agreed upon definition of what will count as waste electronics (C-B).*

## Discussion

This study aimed to examine the application of PPR vs. FPR regulation through impacts on producers and determine both internal and external drivers and barriers for EPR. Accordingly, three important subtopics arose: the state of global e-waste management; the potential of the urban mine; and the role of Extended Producer Responsibility, which were addressed by three research questions: (1) How does physical Extended Producer Responsibility influence producer behavior when compared with financial Extended Producer Responsibility in Japan and Canada’s WEEE recycling management schemes?; (2) How does the difference between Japanese and Canadian systems affect the post-collection cycle of resource recovery and viability of urban mining from e-waste?; (3) What are the drivers and barriers to producers implementing Extended Producer Responsibility in these two systems? These questions will be answered in the following discussion.

### Effects of physical and financial EPR

One finding of this research was that the distinction between physical and financial EPR is not clear in practice. Many interviewees were not familiar with the distinction in the terminology, and although the systems can be differentiated as mentioned, neither case practices mutually exclusive physical or financial EPR. This evokes the bigger issue which plagues the electronic waste field: ambiguity in everything from what actually constitutes electronic waste [11, 48, 49] to whether EPR programs should be classified as physical, financial, collective, individual, or some combination therein, to whether EPR should be the mechanism of choice to drive DfE [4, 33, 50].

In this study the application of physical EPR of the Japanese Home Appliance Recycling Act was found to counteract the most common complaint of collective EPR systems—free-riding [7, 26]. Collective systems are often seen as inducing the group to pay for poor decisions of individual parties [29], but the traceability in a physical responsibility system is posited to have the opposite effect, where the collective can apply pressure to under-performers, who themselves have a vested interest in minimizing cost by improving performance [26]. A more comprehensive classification of EPR systems, according not only to collective vs. individual or physical vs. financial responsibility, but the interplay between all these factors would also be in line with state-of-the-art research, which aims to understand what aspects of EPR may lead to design changes or system improvements [26, 50].

The code *design for recyclability* was used, by interviewees, to address instances of design changes impacting ease of disassembly or recovery at EoL. Concrete examples of regulation impacting the design of products was only mentioned in the Japanese case. This study therefore asserts that physical EPR provides a more direct incentive for producers to improve product design, especially for recyclability, than financial EPR. While not well-covered in existing studies, this aligns with the theory of applying physical responsibility, but is still debated, as it has been asserted that physical responsibility does not apply enough pressure to improve product design [40], and that financial responsibility may result in better collection and recycling rates, which Gupt and Sahay (2015) argue minimizes the need for DfE [12].

In response to the first research question, this study’s findings are that physical EPR as practiced in Japan creates incentive for producers to improve WEEE recycling management practice, therefore promoting resource recovery and recycling process improvements, while financial EPR as applied in the Canadian context incentivizes the collection of e-waste, but the burden of funding the e-waste management system without any resulting financial benefit appears to give priority to cost-saving in upstream processes, therefore negatively affecting EoL management ability.

### Extended producer responsibility, resource recovery, and urban mining

Several interviewees suggested that EPR is effective in driving collection but fails to provide satisfactory outcomes beyond that. This aligns with the literature, as studies have called into question the amount of WEEE (in this case, specifically EU-origin WEEE) showing up in illegal systems abroad, despite improved collection rates reported under the 2012 EU Directive [21, 22]. The Japanese use of a recycling, rather than a collection, target is quite unique to the Japanese system, causing some difficulty in comparison with other regions [51]. These points are important to note because the role-sharing system in Japan means that producers are *not responsible for collection*.

Coupled with the findings that physical recycling responsibility in the Japanese case appears to drive upstream design improvements, namely to improve EoL management, not only the EPR mechanism, but also the lifecycle phase over which producers are responsible is critical to success. This is supported by a study on EU system’s suggests that regulation with a focus on WEEE and WEEE treatment as a waste management issue limits incentive to improve upstream design and incentivize reuse [9]. Similarly, design to simplify recycling may negatively affect material selection or energy inputs during production [52], so the lifecycle stage covered by EPR impacts outcomes, whether environmental- or performance-related.

Interviewees in Canada had little to say on proven design for recyclability initiatives, and in fact, quite the opposite problem—light-weighting—was brought up extensively. The fact that Canada has very few resident producers, coupled with the lack of physical management of recycling systems for Canadian WEEE, suggests that upstream considerations, such as better transport efficiency due to light-weighting, are prioritized over downstream processes that producers are not involved in. While this phenomenon has not been presented in the literature for Canada specifically, it does align with a study on how different implementations of EPR may impact DfE, and how the use of collection over recycling targets (or vice versa) may impact opposite ends of the upstream–downstream lifecycle design [53].

Application of a recycling rate requirement in Japan means that once WEEE enters the formal stream, resources will be extracted and reused; however, the lack of a mandated collection target may disincentivize increasing volume. Although producers in Japan mentioned that the expectation of resource supply from recycling operations may serve as incentive, a study on the state of waste management in Japan suggested that while valuable recycled resources derived from waste have increased modestly, the overall amount of usable material recovered from waste has not seen much improvement since 2004 [54]. A 2019 study also raised this potential for take-back requirements to incentivize recycling items that are still useable as a topic for further research, which very much aligns with the prevalence of the *recycling over reuse* concern raised in Canada [26]. In both Japan and Canada, lack of a clear definition of reuse and a lack of understanding about reuse market size obfuscate efforts to accurately determine real system flows [18].

In both cases, interviewees expressed concern about increasing transboundary regulations limiting the ability for resource trade networks to function. Classification of what qualifies as waste versus resource causes significant consternation in both Japan and Canada and may point to why neither has ratified the stricter regulations of the Basel Ban Amendment, despite being party to the Basel Convention. While the issue of defining e-waste has been well covered, both in the literature and previously in this study [1, 48], there has also been some research on how to more effectively quantify waste vs. resource—whether pre-processed or as second-hand goods—flows in order to limit illegal export while promoting the legitimate international commodity trade [8, 18, 55]. Furthermore, interviewees noted that the Basel Convention has recently introduced stricter measures. While historical issues of informal processing in developing countries remain cause for legitimate concern, there is also a growing body of research exploring the change in regional flows and the potential for positive outcomes under the correct regulatory conditions [56, 57].

### Drivers and barriers for EPR

The findings of this study aligned closely with the literature in case of drivers and barriers for EPR [12, 13, 15]. Table 8 compares the most important drivers in order of importance for producers implementing EPR in the Japanese case, the Canadian case, and two relevant studies which classified the top three drivers similarly. The comparative literature studies were selected for two reasons: first, the Gupt and Sahay (2015) study compared 27 developed OECD, developing OECD, and non-OECD countries utilizing EPR for WEEE management to determine drivers and barriers, and is thus represents a wide breadth of cases. Second, the Zheng et al. (2017) study was selected because it focused on one country—China—therefore, the country-level depth of analysis provided a relevant comparison to this study. External drivers are indicated by and [E], and internal drivers by an [I]. In both Gupt and Sahay ‘s (2015) and Zheng et al. (2017), studies on drivers for EPR, regulation was considered to be the most influential [12, 15]. This was true in both the Japanese and the Canadian case. Reduced cost/financial flows featured in the top three drivers in this research as well as Gupt and Sahay’s (2015) study. The fact that both resource recovery and reduced cost/financial flows were important drivers in the Japanese and Canadian systems, but in opposite order, is of relevance considering the physical vs. financial responsibility EPR comparison. In Japan, resource recovery appeared to be a more important driver, which supports the earlier argument that physical EPR and a mandated recovery rate do positively affect design for recycling considerations. In contrast, reduced cost/financial flows are a greater driver in Canada, which also supports the results—that Canada’s FPR programs have little impact on upstream mitigation of e-waste.

**Table 8:** Classification of importance of top three drivers between cases and literature

Drivers			
Japan	Canada	Gupt and Sahay [12]	Zheng et al. [15]
Regulation/Take-back[E]	Regulation/Take-back[E]	Regulatory Provisions[E]	EPR Regulation[E]
Resource Recovery[E]	Reduced Cost/Financial Flows[I]	Take-back Responsibility[E]	Management Awareness[I]
Reduced Cost/Financial Flows[I]	Resource Recovery[E]	Financial Flows[I]	Corporate Image[E]
	Corporate Image[E]		

*Pre-emptive legislation,* as shown in Table 5, is presented as a new driver not found in previous studies. This aims to encapsulate more broadly producer motivation in responding to EPR, or the idea that producers and industry groups in both Japan and Canada took action to either design the systems that would obligate them in advance of regulation or have collaborated heavily with government in the maintenance and modifications to existing policies. A similar idea is presented by Lifset and Lindhqvist asserting that many cases of DfE have been driven by anticipated legislation, rather than actual [58], but this driver has not previously been presented in EPR discussion as such. The greater prevalence of external drivers in all cases supports the literature findings. Management awareness, as highlighted by Zheng et al., made no significant appearance in this study, but producer and industry representatives were all management-level employees, who were clearly aware of the issue, so this study’s methodology naturally precluded presentation of this driver.

Barriers in the literature were not ranked as clearly as drivers, and the top three listed from two relevant studies in Table 9 were selected and classified according to weight given in each study according to the author of this thesis [13, 14]. This leaves room for interpretation, however both studies measured the relative impact of each barrier, so the interpretation was considered relevant.

**Table 9:** Classification of importance of top three barriers between cases and literature

Barriers			
Japan	Canada	Evans, Vermeulen [13]	Yu et al. [14]
Cost[I]	Complex Legislation[E]	Free Riders[E]	Cost[E]
Illegal Collection[E]	Lack of Harmonization[E]	Illegal Export[E]	Lack of Regulation Pressure[E]
No Incentive to Improve[I]	Cost[I]	Cost[I]	Lack of Market Demand[E]
Complex Legislation[E]			

Both cases and studies had cost as one of the top three barriers, reinforcing the driver findings that producer participation in EPR is firstly due to regulation, and therefore cost to participate is the resulting barrier. As this study found, and as seen previously in Table 7, both Japan and Canada suffer from complex legislation—both in terms of obligating scope under multiple different systems and assigning responsibility.

The second barriers in each case are an important finding: in the case of Japan, illegal collection, and associated dumping or export, was a concern explicitly brought up by every interviewee, and also supported by the available literature [42, 54, 59]. Much like the drivers, the barriers to producers implementing sufficient EPR practices are mostly external rather than internal. The highlight of cost as a barrier, and reduced cost/financial flows as a driver may lend weight to the consideration of financially congratulatory vs. punishing compliance mechanisms—a salient topic of discussion in the literature [33, 60].

This study also presented a new barrier, which is *no incentive to improve*. While Yu et al. presented a related barrier, *lack of regulation pressure* [14], that is an external barrier which aligns more closely with this study’s *ineffective targets*. *No incentive to improve* refers holistically to an internal condition where producers see no measurable benefit to improving performance beyond minimum compliance. This apathy or stagnation of systems is not new to the literature [52], but has not been framed from the producer perspective in this way before.

## Conclusion

This study is, to authors knowledge, the only available study which compares two different country cases through the lens of physical vs. financial EPR. It also highlights how physical and financial EPR may influence different parts of the upstream vs. downstream mitigation process.

The findings of this study in response to the three stated research questions are as follows:

1. Physical EPR in Japan was suggested to drive upstream design improvements to EEE which result in simplified processing of WEEE and financial EPR in the Canadian case apparently promotes upstream cost-savings which negatively affect downstream processing.
2. While physical EPR and the regulatory requirements of the Japanese system required producers to put a greater focus on resource recovery than the financial EPR requirements of the Canadian system, there are also external factors—most notably the disparity between Japan and Canada’s natural resource availability, which currently make the urban mine more viable in Japan.
3. The drivers and barriers found in this study for both systems align with current knowledge on EPR—in both cases, the greatest driver to producers implementing EPR is simply that they are legally obligated to so. However, the greatest barriers differed—highlighting that the Japanese case must contend with stringent physical responsibilities which carry a heavy associated cost, while in the Canadian case, contending with three different types of legislation, even though all effectively prescribe financial EPR, create a complex system that producers struggle to adhere to, minimizing their ability to improve WEEE management.

This study sheds some light on various EPR regulatory methods in practice and how they are applied in two countries that are vastly different but share the same supranational obligations. The similarities, particularly in the resulting drivers and barriers, indicates that producers face comparable motivations and challenges across markets. This carries implications for policymakers, suggesting that harmonization of policy could improve global management of WEEE, even as certain policy designs must be specific to each country or region—as noted, Japan’s resource recovery system is self-sustaining, while in Canada, without more stringent policies, resource recovery potential is more aspirational than practical. Furthermore, this study presented new drivers and barriers, namely *pre-emptive legislation* and *no incentive to improve,* which have not been explicitly addressed in past studies on EPR in electronic waste management, and thus could provide the basis for future study. Furthermore, as has been discussed, while the implementation methodology of EPR is important, this study has reinforced the oft-addressed issue of policy in general, and the Basel Convention specifically; the categorization of WEEE is inconsistent across regions, creating challenges both for national or regional policymakers and for producers trying to be compliant across regions.

In Japan, the impact of the fee structure and its relationship to the issue of illegal collection and export would be an interesting topic, but there is currently a lack of available English-language research to drive future study. For the Canadian case, future research on Ontario’s new IPR system may provide more concrete data to study, currently distinctly lacking—it could also serve as a comparative counterpoint to the prevailing collective EPR frameworks present in Canada. Furthermore, these specific topics as well as drivers and barriers from this study are relevant to policymakers from other countries implementing EPR regulation. For EPR in e-waste in general, comprehensive classification on how it is applied would provide useful discourse into how it impacts producer practice—as this study and a literature comparison showed, even across regulatory regions and EPR systems, drivers and barriers for producers were relatively comparable. Finally, the current discrepancy between classification systems used—such as physical, financial, individual, and collective—serve only to dilute the distinction between downstream vs. upstream impacts of EPR and limit comparability between policies. In addition, how the differing governance systems for policy implementation between Japan and Canada, as mentioned briefly in Sect. 3.1, affect EPR system performance could bring to light other drivers and barriers for producers in each of the case countries, or in a comparison with other regions. Future research on how specific policy measures drive change at which lifecycle phase may provide greater insight into the true effectiveness of EPR in improving e-waste management.

## Data Availability

Basic interview framework presented in Appendix, other data available upon reasonable request to corresponding author.

## Appendix

### Standard interview frameworks

#### Producer/industry representatives—policy & practice, drivers & barriers

1. How do you define e-waste?
2. How do you define and practice EPR?
3. Do you have any repair or re-sale initiatives?
4. Can you briefly describe the lifespan and stages your products go through?
  - Who are the key players/stakeholders in these stages?
5. Do you manage end-of-life waste with physical responsibilities (take-back, collection, recycling) or fiscal (contracted take-back, collection, etc.)?
6. Can you tell me about your policies regarding end-of-life electronic waste that originates with you?
7. How long have these policies been in place, and how have they changed over time?
8. Is your EPR policy guided by legislation (top-down), or internal initiative?
9. Who do you think bears the cost of compliance with EPR legislation?
10. Have you seen measurable benefits with the implementation of your EPR policies?
11. How does the design or production process for new electronics take the post-use cycle into account?
12. Can you discuss some unforeseen positive and/or negative side effects of implementing EPR?
13. Do you feel that existing legislation around electronic waste is effective in reducing improperly treated e-waste?
14. How would you improve or change it?
15. Do you take any steps to ensure that WEEE originating with you doesn’t end up in unknown streams?
16. Do you have a process for verifying disposal?
17. What do you see as the current barriers to improving e-waste management?

What about drivers in improving e-waste management?

#### Regulation—Governing bodies/NGOs

1. What is the existing legislation on e-waste and how recent is it?
2. Has it changed since it was first introduced? If so, how?
3. Can you describe any meaningful or measurable improvements in e-waste management you may have seen under current legislation?
4. What common issues are you seeing with the current situation (i.e. non-compliance, lack of data, etc.)?
5. How do you define stakeholder roles in the e-waste management scenario (producers/importers, consumers, governing officials, recyclers)?
6. Do you feel there is currently enough accurate and reliable data available to assess the effectiveness of e-waste management practice?
7. What are the key issues or blind spots you see with the current system, and what about the system works effectively?
8. How do you measure the success of e-waste management regulation currently in place?
9. Is data comparable to other provincial or international sources?
